# Sweroside Protects Against Myocardial Ischemia–Reperfusion Injury by Inhibiting Oxidative Stress and Pyroptosis Partially via Modulation of the Keap1/Nrf2 Axis

**DOI:** 10.3389/fcvm.2021.650368

**Published:** 2021-03-19

**Authors:** Jun Li, Cuiting Zhao, Qing Zhu, Yonghuai Wang, Guangyuan Li, Xinxin Li, Yuhong Li, Nan Wu, Chunyan Ma

**Affiliations:** ^1^Department of Cardiovascular Ultrasound, The First Affiliated Hospital of China Medical University, Shenyang, China; ^2^Department of Ultrasound, The First Affiliated Hospital of Jinzhou Medical University, Jinzhou, China; ^3^The Core Laboratory of the First Affiliated Hospital of China Medical University, Shenyang, China

**Keywords:** myocardial ischemia reperfusion injury, sweroside, oxidative stress, pyroptosis, nuclear factor E2-associated factor 2

## Abstract

**Aims:** Sweroside, a secoiridoid glucoside extracted from *Swertia pseudochinensis* Hara, is reported to possess antioxidant and anti-inflammatory activities. However, whether sweroside has a protective effect on myocardial ischemia–reperfusion (IR) injury is yet to be elucidated. The present study aimed to confirm the cardioprotective effect of sweroside and to identify its underlying mechanism.

**Methods and Results:** H9c2 cells were pretreated with sweroside and then underwent hypoxia–reoxygenation. Cell Counting Kit-8, creatine kinase-myocardial band (CK-MB) and lactate dehydrogenase (LDH) assays were conducted to detect cell viability and myocardial injury, respectively. The Langendorff method was used to induce myocardial IR injury *ex vivo*. Triphenyltetrazolium chloride staining was performed to detect myocardial infarct size, while protein expression was analyzed using western blotting. Overall, the results indicated that sweroside pretreatment dose-dependently led to a significant enhancement in cell viability, a decrease in release of CK-MB and LDH, a reduction in infarct size, and an improvement in cardiac function. Additionally, sweroside pretreatment caused a marked suppression of oxidative stress, as evidenced by the fact that sweroside decreased the accumulation of reactive oxygen species and malondialdehyde, while enhancing the activities of superoxide dismutase and glutathione peroxidase. Moreover, sweroside was found to notably repress pyroptosis, as sweroside blocked pore formation in the cell membrane, inhibited caspase-1 and interleukin (IL)-1β activity, and decreased the expression levels of NLR family pyrin domain containing 3 (NLRP3), apoptosis-associated speck-like protein containing a CARD, cleaved caspase-1, and IL-1β. Mechanistically, it was found that sweroside inhibited Kelch-like ECH-associated protein 1 (Keap1) and induced nuclear factor E2-associated factor 2 (Nrf2) nuclear translocation. Furthermore, the inhibition of oxidative stress and pyroptosis by sweroside could be abrogated via the inhibition of Nrf2 expression, which suggested that the protective effect induced by sweroside was Nrf2-dependent.

**Conclusions:** The present study demonstrated that sweroside pretreatment could protect against myocardial IR injury by inhibiting of oxidative stress and NLRP3 inflammasome-mediated pyroptosis partially via modulation of the Keap1/Nrf2 axis.

## Introduction

Ischemic heart disease (IHD), a major cause of mortality and disability worldwide, is estimated to account for ~7.4 million deaths globally per year (1). Although myocardial reperfusion therapy, typically represented by percutaneous coronary intervention and coronary artery bypass grafting, has been the mainstream approach for IHD treatment, ischemia–reperfusion (IR) injury remains an unsolved problem that mainly impacts the effectiveness of reperfusion therapy (2). Therefore, how to effectively prevent myocardial IR injury has gained increased interest from researchers.

While the mechanism of myocardial IR injury has not been fully elucidated, oxidative stress (3) and inflammation (4) have proved to be responsible for myocardial IR injury. Nuclear factor E2-associated factor 2 (Nrf2) is a key transcription factor that serves an important role in the regulation of oxidative stress (5) and inflammation (6). In a physiological state, Nrf2 mainly resides in the cytoplasm by binding to Kelch-like ECH-associated protein 1 (Keap1), which is a physiological inhibitor of Nrf2. However, under an oxidative stress condition, Nrf2 translocates into the nucleus by separating from Keap1 and subsequently activates the transcription of antioxidant genes, such as heme oxygenase (HO)-1, which protects cells against oxidative stress- and inflammation-induced injury (7, 8). For example, geniposide-induced preconditioning alleviates myocardial IR injury by activating the Nrf2/HO-1 signaling pathway to inhibit oxidative stress (9). Moreover, activation of Nrf2 can repress NLR family pyrin domain containing 3 (NLRP3) inflammasome-mediated pyroptosis via modulation of reactive oxygen species (ROS) in cerebral IR injury (10, 11). Therefore, Nrf2 is considered as a therapeutic target for myocardial IR injury (12).

**Figure 1:**
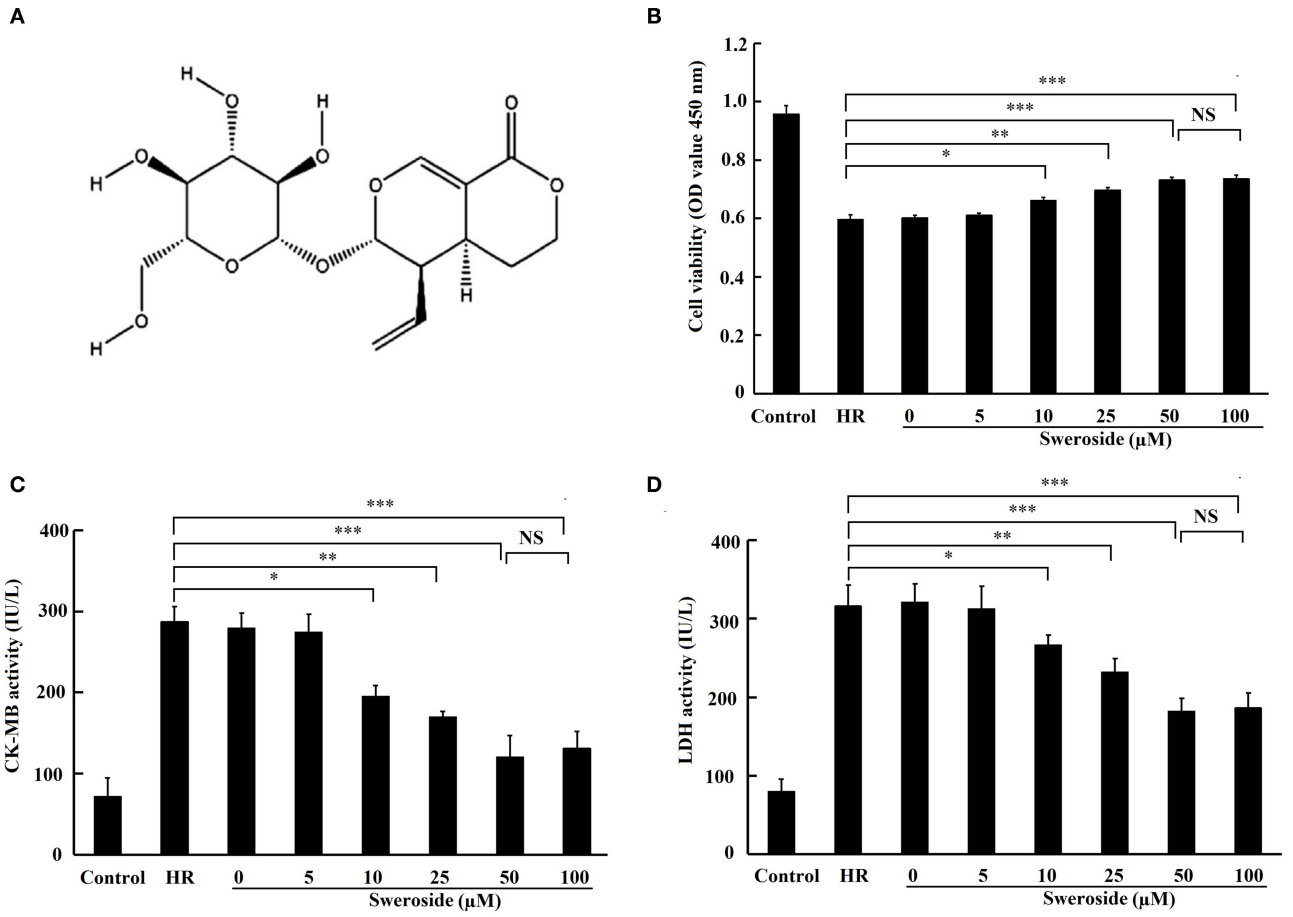
The protective effect of sweroside on hypoxia/reoxygenation (HR)-induced myocardial injury *in vitro*. H9c2 cells were pretreated with a various concentrations of sweroside for 24 h and then underwent HR. **(A)** The chemical structure of sweroside. **(B)** Cell viability was assessed using Cell Counting Kit-8 assay. **(C,D)** Detection of creatine kinase isoenzyme-myocardial band (CK-MB) and lactate dehydrogenase (LDH) levels in culture medium. Each cell experiment was repeated at least three times. *P < 0.05; **P < 0.01; ***P < 0.001; NS, not significant.

Accumulating evidence has revealed that some active constituents of traditional Chinese medicine, such as ginsenoside (13, 14) and baicalin (15, 16), could protect against myocardial IR injury via their antioxidant and anti-inflammatory activities. Thus, traditional Chinese medicine provides a novel strategy for the treatment of myocardial IR injury (17). It has been reported that sweroside, a secoiridoid glucoside extracted from the plant *Swertia pseudochinensis* Hara (Figure 1A), possesses powerful antioxidant (18) and anti-inflammatory activities (19, 20). For instance, Ma et al. suggest that sweroside pretreatment inhibited aconitine-trigged oxidative stress and intercellular ROS production in cardiomyocytes (18). It also has been found that sweroside could prevent against lipopolysaccharide (LPS) or interleukin (IL)-1β-induced inflammation via suppression of NF-kappaB (NF-κB) signaling pathway (19, 20). Notably, a recent study has demonstrated that sweroside could lead to suppression of NLRP3 inflammasome activation (21). However, the detailed mechanism has not yet been elucidated. Moreover, as predicted by Molecular Operating Environment (MOE) software, sweroside may interact with Keap1. Hence, we hypothesized that sweroside may exert a protective effect on myocardial IR injury via its antioxidant and anti-inflammatory activities via the Keap1/Nrf2 axis.

In the present study, we tested this hypothesis in *in vitro* and *ex vivo* models. The results indicated that sweroside pretreatment alleviated the extent of myocardial IR injury *in vitro* and *ex vivo*, which involved the repression of oxidative stress and NLRP3 inflammasome-mediated pyroptosis partially via modulation of the Keap1/Nrf2 axis.

## Materials and Methods

### Cell Culture

Rat myocardial cells (H9c2 cell line) were obtained from the Shanghai Institutes for Biological Sciences (Shanghai, China) and were routinely cultivated in Dulbecco's modified Eagle medium (DMEM) supplemented with 10% fetal bovine serum (FBS) under the conditions of 37°C and 5% CO_2_.

### Hypoxia/Reoxygenation Model

The hypoxia/reoxygenation (HR) model was established as described previously (22). Specifically, when the cell density reached about 70%, cells were relocated to a tri-gas incubator with an atmosphere of 94% N_2_, 5% CO_2_, and 1% O_2_ and subsequently cultured with Earle's medium without glucose and FBS for 6 h to establish hypoxia. At the end of the incubation, the medium was replaced with DMEM supplemented with 10% FBS, and cells were transferred to CO_2_ incubator with an atmosphere of 5% CO_2_ for 3-h reoxygenation.

### Cell Treatment

Sweroside (CAS no. 14215-86-2; purity ≥98%) was purchased from Meilun Biochemical Co., Ltd. (Dalin, China). Sweroside was dissolved in dimethyl sulfoxide (DMSO) (Sigma-Aldrich; Merck KGaA) and further diluted with culture medium until the DMSO concentration was <0.1%. Cells were pretreated with various concentrations of sweroside (0–100 μM) for 24 h and then underwent HR.

### Cell Transfection

Small interfering (si) RNA targeting Nrf2 and its negative control were designed and synthesized by Guangzhou RiboBio Co., Ltd. (Guangzhou, China) and were then transfected into cells using Lipofectamine® 2000 (Invitrogen; Thermo Fisher Scientific, Inc., Waltham, MA, USA) according to the manufacturer's instructions. Following transfection for 24 h, cells were further processed for sweroside treatment, followed by HR.

### Cell Viability Assay

Cells were seeded into 96-well plates at a concentration of 5,000 cells/well, treated with sweroside, and subsequently underwent HR. A Cell Counting Kit (CCK)-8 assay (Dojindo Molecular Technologies, Inc., Kumamoto, Japan) was performed to evaluate cell viability, following the manufacturer's instructions. The absorbance was measured at 450 nm on a microplate reader.

### Myocardial Enzyme Tests

Lactate dehydrogenase (LDH) and creatine kinase-myocardial band (CK-MB) are known as myocardial injury markers (23). Hence, the levels of LDH and CK-MB in the culture medium were measured to evaluate the extent of myocardial injury using LDH and CK-MB assay kits, respectively (provided by Jiancheng Bioengineering Institute, Nanjing, China), following the manufacturer's instructions.

### Measurement of Reactive Oxygen Species Content

The cells were cultured on a small glass sheet and fixed with 4% paraformaldehyde at room temperature for 15 min. After being washed three times with phosphate-buffered saline (PBS), cells were incubated with 2′,7′-dichlorodihydrofluorescein diacetate (DCFH-DA), which acts as a sensitive ROS probe (Jiancheng Bioengineering Institute, Nanjing, China), at 37°C for 1 h. DCFH-DA-stained cardiomyocytes were observed and imaged under a fluorescence microscope.

### Determination of Malondialdehyde, Superoxide Dismutase, and Glutathione Peroxidase Enzymes

After being digested by trypsin, cells were collected and subsequently resuspended in 0.5 ml of PBS prior to being comminuted with an ultrasonic disintegrator. Cell homogenates were centrifugated at 12,000 × g for 10 min, followed by collection of the supernatant. The protein concentration of the supernatant was determined using an enhanced bicinchoninic acid (BCA) Protein Assay kit (Beyotime Institute of Biotechnology, Haimen, China) following the manufacturer's guidelines. The malondialdehyde (MDA) content, and the activities of superoxide dismutase (SOD) and glutathione peroxidase (GSH-Px) were measured using MDA, SOD, and GSH-Px detection kits (all from Jiancheng Bioengineering Institute, Nanjing, China), respectively, following the manufacturer's instructions.

### Detection of Cell Membrane Integrity

Pyroptosis is characterized by pore formation in the cell membrane (24). Thus, propidium iodide (PI) may permeate into pyroptotic cells and stain nucleus due to the loss of cell membrane integrity. For this experiment, cells were incubated with PI (10 μM) for 30 min at room temperature, followed by re-staining with 4′,6-diamino-2-phenyl indole (DAPI). The results of the staining were observed under a fluorescence microscope.

### Measurement of Caspase-1 Activity

After treatment, cells were digested with pancreatic enzymes and collected. The activity of caspase-1 in the collected cells was detected using a caspase-1 activity assay kit (Beyotime Institute of Biotechnology) following the manufacturer's instructions.

### Measurement of Interleukin-1β Concentration

The concentration of IL-1β in the culture medium was measured using an enzyme-linked immunosorbent assay (ELISA) with the Rat IL-1β ELISA kit (MULTY SCIENCE) according to the manufacturer's instruction.

### Molecular Docking Simulation

The binding between sweroside and Keap1 was stimulated using MOE software. Information regarding the Keap1 protein was downloaded from the RCSB Protein Data Bank (http://www.rcsb.org/pdb/home/home.do), and an sdf format of sweroside was obtained from ChemDraw version 19.0. Sweroside and the molecular structure of Keap1 were protonated in 3D at 300°C, pH = 7 and with minimized energy using MOE software. Sweroside was docked to active sites of Keap1 using the Triangle Matcher placement and Induced Fit refinement methods.

### Immunofluorescence Staining

Cells were placed on glass slides, fixed with 4% paraformaldehyde for 15 min, permeabilized with 0.1% Triton X-100 for 30 min, and then blocked with 5% goat serum for 15 min at room temperature. Subsequently, cells were incubated with diluted primary antibody against Nrf2 (1:200; cat. no. ab89443; Abcam, Hong Kong) at 4°C overnight, followed by incubation with Cy3-labeled goat antimouse IgG (1:2,000; cat. no. 33208ES60; Yeason, Shanghai, China) for 60 min at room temperature. Nuclei were re-stained with DAPI, and the results of the staining process were observed under a fluorescence microscope.

### Animals

The animals used in the present study were healthy male Wistar rats (age, 8 weeks; weight, 240–260 g), which were purchased from the Animal Laboratory Center of China Medical University (Shenyang, China). The rats were kept at 22–24°C under 12-h daylight:12-h dark conditions and had free access to clean water. The body weight of the rats was monitored twice a week. The use of animals in the present study was authorized by the Institutional Animal Care and Use Committee of China Medical University, and the Guide for the Care and Use of Laboratory Animals [National Institutes of Health (NIH), USA] was followed closely.

### Establishment of the Isolated Rat Heart Ischemia–Reperfusion Model

Animal anesthesia was induced via an intraperitoneal injection of sodium pentobarbital (30 mg/kg) (22). The rat heart was isolated from the thoracic cavity, hung on a Langendorff perfusion device from the root of the aorta, and perfused with O_2_-saturated Krebs–Henseleit (K-H) solution (127 mmol/L of NaCl, 17.7 mmol/L of NaHCO_3_, 5.1 mmol/L of KCl, 1.5 mmol/L of CaCl_2_, 1.26 mmol/L of MgCl_2_, and 11 mmol/L of d-glucose, pH = 7.4) at a constant pressure of 75 mmHg and in a constant temperature (37°C), as described previously (22). Myocardial IR was induced by stopping K-H solution perfusion for a period of 30 min, followed by reperfusion with K-H solution for 90 min. The fluid-filled latex balloon, which was connected to a pressure sensor, was inserted into the left ventricle to dynamically monitor the alteration of cardiac function using a homodynamic system (MP150; BIOPAC Systems, Inc.; Goleta, CA, USA).

### Animal Treatment and Experimental Groups

Sweroside at the dose of 25, 50, and 100 mg/kg was intraperitoneally injected into rats once a day for 5 consecutive days before myocardial IR injury was induced. The dose of sweroside used in this study was based on a previous study (18). A total of 48 rats were separated equally into six groups (*n* = 8), as follows: (i) the control group, the isolated heart underwent 120 min of perfusion without interruption; (ii) the IR group, the isolated heart was subjected to interruption of perfusion for 30 min, followed by reperfusion for 120 min; (iii) the vehicle group, the rats received an intraperitoneal injection of 1 ml of saline once a day for 5 consecutive days prior to heart operation, followed by IR, as described for the IR group; (iv) the 25 mg/kg sweroside treatment group, the rats received an intraperitoneal injection of sweroside (25 mg/kg) once a day for 5 consecutive days before heart isolation, followed by IR, as described for the IR group; (v) the 50 mg/kg sweroside treatment group, the rats received 50 mg/kg of sweroside treatment and underwent IR, as described for the 25 mg/kg sweroside group; and (vi) the 100 mg/kg sweroside treatment group, the rats received 50 mg/kg of sweroside treatment and underwent IR as described for the 25 mg/kg sweroside group.

### Measurement of Infarct Size

At the end of the reperfusion, the hearts were detached from the device, refrigerated at −20°C for 1 h, and then cut into 1-mm-thick sections. These sections were stained with triphenyltetrazolium chloride solution, as described previously (22).

### Western Blotting

Total proteins were obtained from the collected cells using radioimmunoprecipitation assay (RIPA) buffer. Nuclear and cytoplasmic proteins were extracted using a Nuclear and Cytoplasmic Protein Extraction kit (Beyotime Institute of Biotechnology, Shanghai, China), and the protein concentration was determined using an enhanced BCA Protein Assay kit (Beyotime Institute of Biotechnology), in accordance with the manufacturer's instructions. Protein samples were thermally denatured, separated by 8 or 10% sodium dodecyl sulfate–polyacrylamide gel electrophoresis (SDS-PAGE) electrophoresis, transferred to polyvinylidene difluoride (PVDF), membranes and blocked with 1% bovine serum albumin for 1 h at room temperature. Then, membranes were incubated with diluted primary antibodies, including anti-Nrf2 (cat. no. WL02135), anti-HO-1 (cat. no. WL02400), anti-Keap1 (cat. no. WL03285), anti-NLRP3 (cat. no. WL02635), anti-associated speck-like protein containing a CARD (ASC; cat. no. WL02462), anti-cleaved-IL-1β (cat. no. WL00891), and anti-cleaved caspase-1 (cat. no. WL03450) (all antibodies diluted 1:1,000 and purchased from Wanleibio Co., Ltd, Shenyang, China) at 4°C overnight. After being washed three times with PBS, the membranes were incubated with horseradish peroxidase-labeled IgG (1:5,000; Zhongshan Jinqiao Biotechnology, Beijing, China) at room temperature for 30 min. The membranes were then developed using a BeyoECL Plus kit (Beyotime Institute of Biotechnology) following the operation guideline. Image J2x analysis software (NIH, Bethesda, MD, USA) was used to analyze relative densitometry values.

### Statistical Analysis

Data are presented as the mean ± standard deviation. The statistical significance between groups was determined using one-way analysis of variance, followed by the least significant difference *post-hoc* test. SPSS version 17.0 software (SPSS, Inc., Chicago, IL, USA) was employed to conduct all statistical analyses, and *P* < 0.05 was considered to indicate a statistically significant difference.

## Results

### Sweroside Pretreatment Alleviated Hypoxia/Reoxygenation-Induced Myocardial Injury *in vitro*

The results demonstrated that cell viability was remarkably enhanced (Figure 1B; 10, 25, 50, and 100 μM of sweroside vs. HR: 0.652 ± 0.009, 0.697 ± 0.007, 0.732 ± 0.009, 0.735 ± 0.011 vs. 0.597 ± 0.016), and the release of CK-MB and LDH (Figures 1C,D) was significantly decreased by 10, 25, 50, and 100 μM of sweroside pretreatment. Moreover, the 25 μM sweroside treatment group displayed higher cardioprotection than the 10 μM sweroside treatment group, while the 50 μM sweroside treatment group had an improved cardioprotection than the 25 μM sweroside treatment group. However, no additive protective effect was found in the 100 μM sweroside treatment group compared with the 50 μM sweroside treatment group. Therefore, these results suggested that sweroside pretreatment dose-dependently alleviated HR-induced myocardial injury *in vitro*, and 50 μM was used for subsequent experiments.

### Sweroside Pretreatment Ameliorated Ischemia–Reperfusion-Induced Myocardial Injury *ex vivo*

**Figure 2:**
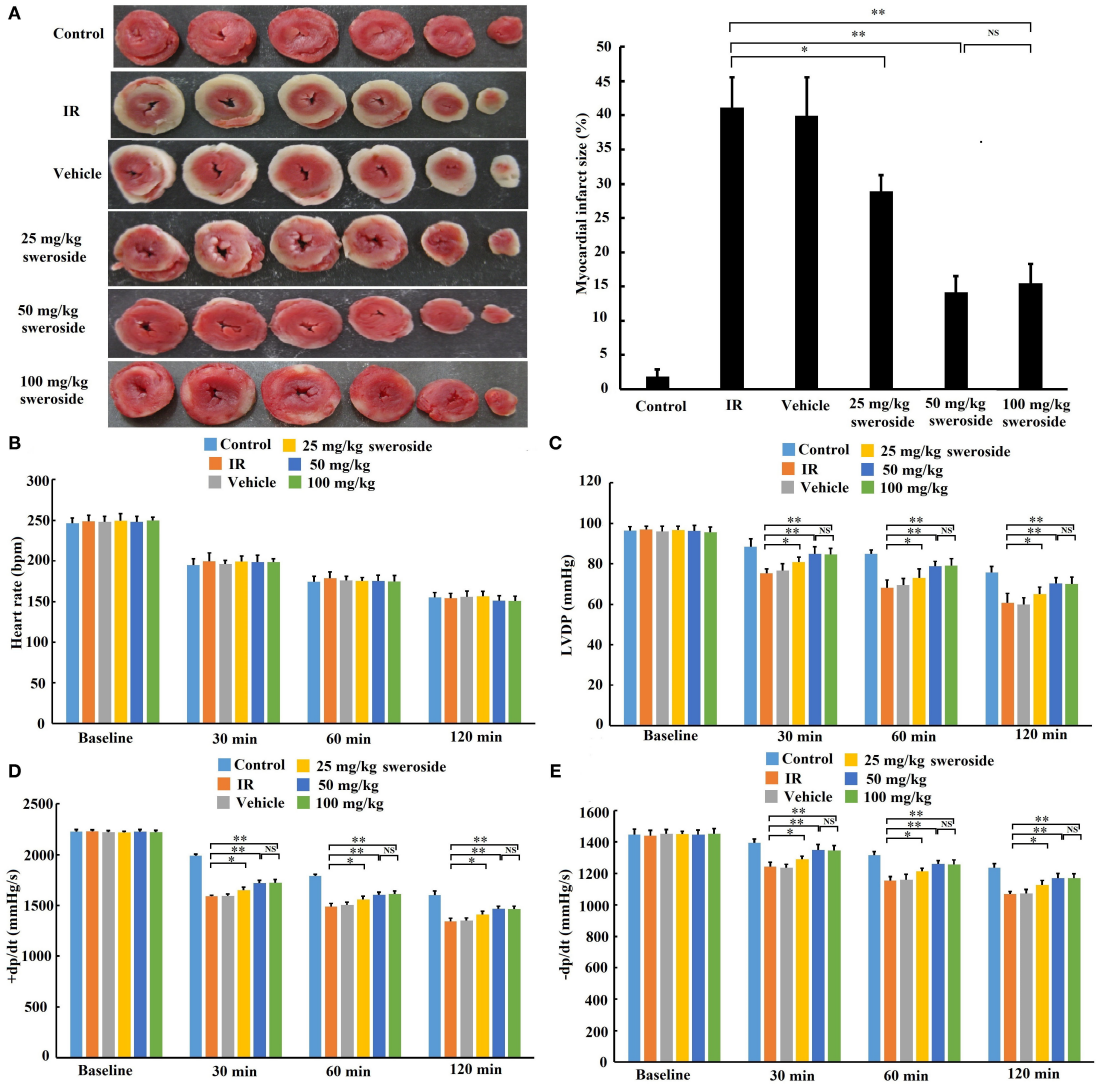
The protective effect of sweroside on ischemia–reperfusion (IR)-induced myocardial injury *ex vivo*. After pretreatment with various doses of sweroside, the rat heart was isolated and then subjected to IR. **(A)** Determination of myocardial infarct size using TTC staining. **(B–E)** Comparison of the difference in heart rate, left ventricular developed pressure (LVDP), positive first-order derivative of ventricular pressure (+dp/dt), and negative first-order derivative of ventricular pressure (–dp/dt) between groups. Data are presented as the mean ± standard deviation, *n* = 6. *P < 0.05; **P < 0.01; NS, not significant.

Myocardial infarct size in the sweroside treatment group (25, 50, and 100 mg/kg) was significantly decreased compared with that in the IR group (25, 50, and 100 mg/kg of sweroside vs. IR: 28.90 ± 2.35%, 14.14 ± 2.38%, 15.51 ± 2.78% vs. 41.14 ± 4.37%). Moreover, the infarct size in the 50 mg/kg sweroside treatment group was small compared with that in the 25 mg/kg sweroside treatment group. However, there was no significant difference in the infarct size between the 50 mg/kg sweroside treatment group and the 100 mg/kg sweroside treatment group (Figure 2A). In addition, the left ventricular developed pressure (LVDP) (Figure 2C) and positive/negative first-order derivative of ventricular pressure (±dp/dt) (Figures 2D,E) values in the sweroside treatment group (25, 50, and 100 mg/kg) were significantly increased compared with those in the IR group, although there was no significant difference in the heart rate (Figure 2B). Similar to the result of the infarct size, the LVDP and ±dp/dt values in the 50 mg/kg sweroside treatment group were higher compared with those in the 25 mg/kg sweroside treatment group, but these showed no significant difference as compared with the 100 mg/kg sweroside treatment group. Taken together, these results suggested that sweroside decreased myocardial infarct size and ameliorated cardiac function in a dose-dependent manner and that 50 mg/kg of sweroside treatment induced the best cardioprotection *ex vivo*.

### Sweroside Inhibited Hypoxia/Reoxygenation-Induced Oxidative Stress

**Figure 3:**
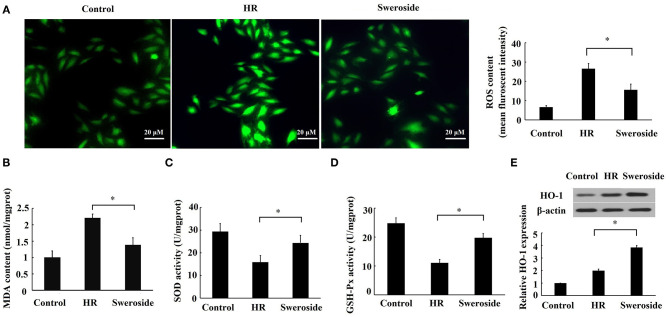
Sweroside inhibited hypoxia/reoxygenation (HR)-induced oxidative stress. H9c2 cells were pretreated with 50 μM of sweroside for 24 h and then underwent HR. **(A)** Reactive oxygen species (ROS) in cells was detected using 2′,7′-dichlorodihydrofluorescein diacetate (DCFH-DA). **(B)** Measurement of malondialdehyde (MDA) content. **(C)** Measurement of superoxide dismutase (SOD) activity. **(D)** Measurement of glutathione peroxidase (GSH-Px) activity. **(E)** HO-1 level detected via western blotting. Each cell experiment was repeated at least three times. *P < 0.05.

The level of ROS, MDA, SOD, and GSH-Px can reflect cellular oxidative stress (25). Sweroside pretreatment significantly attenuated the levels of ROS and MDA content (Figures 3A,B) that were induced by HR, while it enhanced the activities of SOD and GSH-Px (Figures 3C,D) that were repressed by HR. HO-1 is a major antioxidant molecule, and the level of HO-1 can also reflect the status of oxidative stress (26). It was found that sweroside pretreatment led a nearly two-fold increase in the expression of HO-1 (Figure 3E). Collectively, these results suggested that sweroside could inhibit HR-induced oxidative stress.

### Sweroside Inhibited Hypoxia/Reoxygenation-Induced Pyroptosis

**Figure 4:**
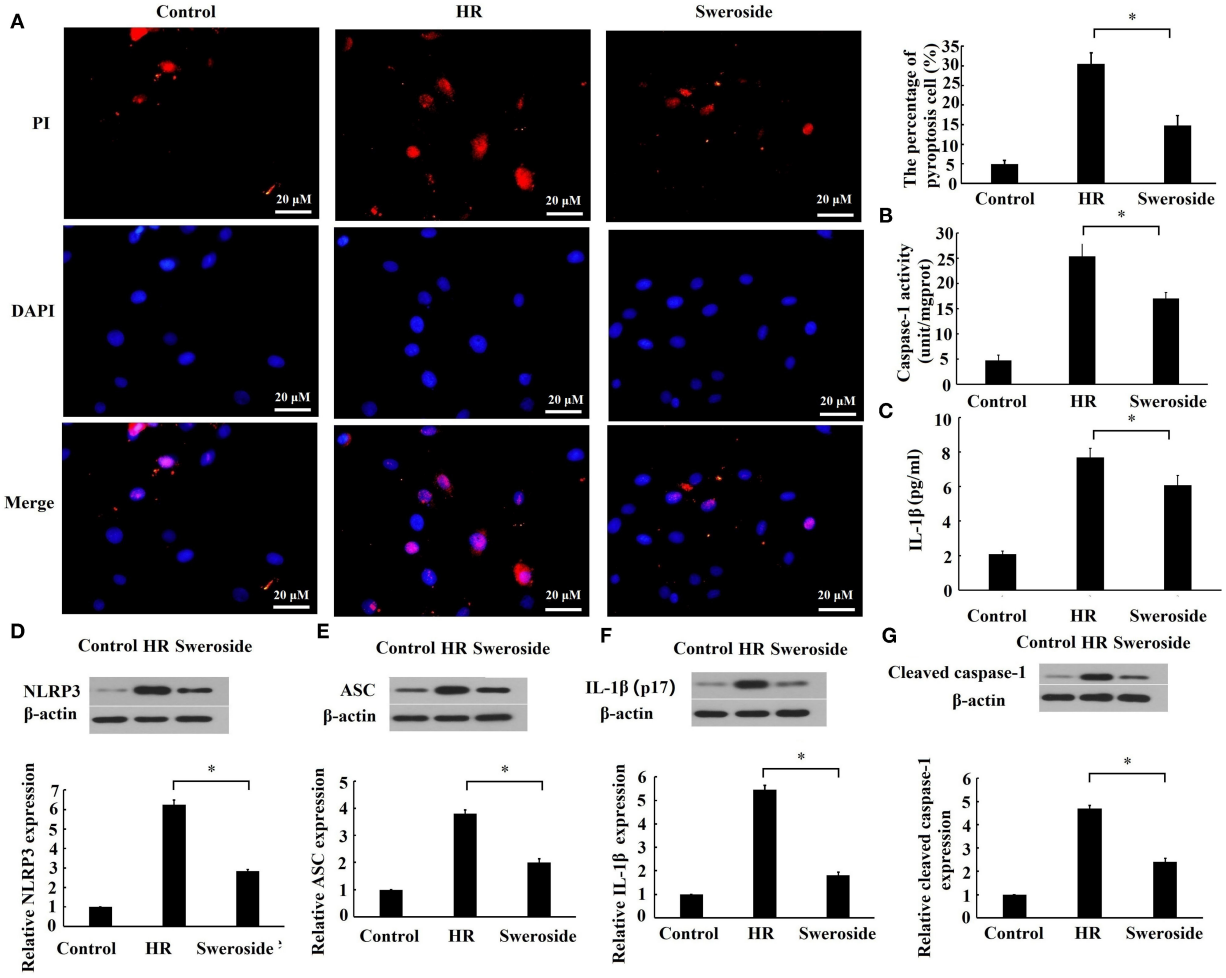
Sweroside inhibited hypoxia/reoxygenation (HR)-induced pyroptosis. H9c2 cells were pretreated with 50 μM of sweroside for 24 h and then underwent HR. **(A)** Pyroptosis cell was characterized by pore formation in the cell membrane. The cell nucleus stained by propidium iodide (PI) indicated a pyroptotic cell. **(B)** The activity of caspase-1 and **(C)** the level of IL-1β in culture medium were detected using enzyme-linked immunosorbent assay (ELISA). Western blotting was performed to detect the expression levels of NLRP3 **(D)**, ASC **(E)**, IL-1β **(F)**, and cleaved caspase-1 **(G)**. Each cell experiment was repeated at least three times. *P < 0.05.

To evaluate the effect of sweroside on pyroptosis, the cell membrane integrity, the activities of caspase-1 and IL-1β, and pyroptosis-related proteins were detected. As shown in Figure 4A, sweroside pretreatment markedly prevented the loss of cell membrane integrity induced by HR, and the percent of pyroptotic cells was reduced by sweroside pretreatment (sweroside vs. HR: 14.76 ± 2.58% vs. 30.44 ± 2.82%). Moreover, the activities of caspase-1 and IL-1β in culture medium were significantly decreased by sweroside pretreatment (Figures 4B,C). The expression levels of NLRP3, ASC, IL-1β, and cleaved caspase-1 were also decreased by 55, 48, 65, and 49% by sweroside pretreatment, respectively (Figures 4D–G). Altogether, these results indicated that sweroside inhibited HR-induced pyroptosis.

### Sweroside Repressed Keap1 and Promoted Nuclear Translocation of Nrf2

**Figure 5:**
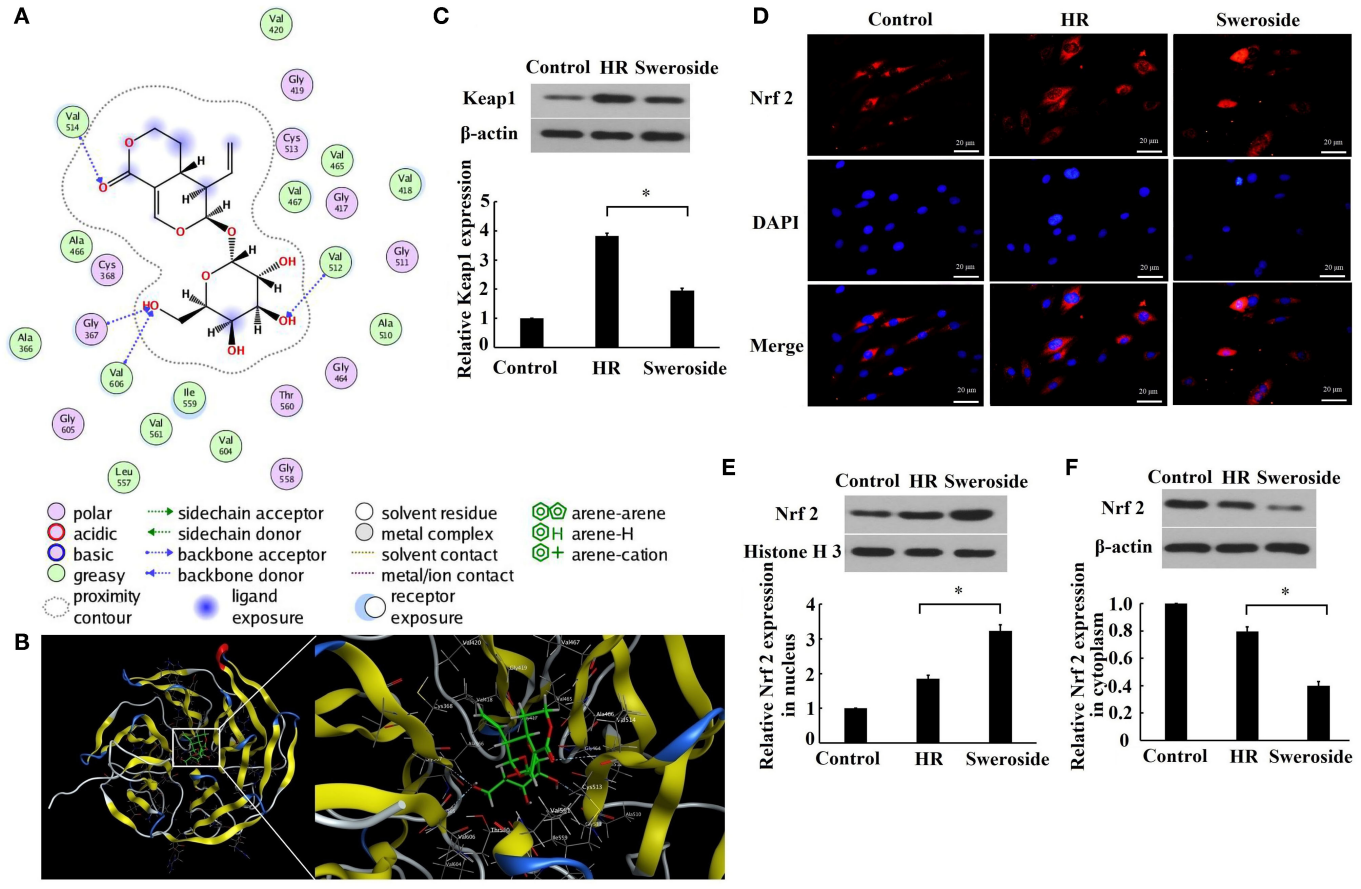
Sweroside repressed Keap1 and promoted nuclear translocation of Nrf2. **(A)** 2D diagrams for the complex between sweroside and Keap1 was simulated using Molecular Operating Environment (MOE). The chemical structure of sweroside is shown in the center, surrounded with the key interacting amino acids. **(B)** 3D models of the docking conformation between sweroside and Keap1. H9c2 cells were pretreated with 50 μM of sweroside for 24 h and then underwent hypoxia/reoxygenation (HR). **(C)** Western blot analysis of Keap1 expression. **(D)** Nrf2 nuclear translocation was observed via immunofluorescence staining. Scale bar = 20 μm. **(E,F)** Western blot analysis of the expression level of Nrf2 in the cytoplasm and nuclei. Each cell experiment was repeated at least three times. *P < 0.05.

As predicted by the molecular docking model, it was found that sweroside may interact with Keap1 (Figures 5A,B). Furthermore, it was identified that the expression level of Keap1 was significantly decreased by 51% after sweroside treatment (Figure 5C). The results demonstrated that the expression level of Nrf2 in the nucleus was nearly enhanced two-fold, while the level of Nrf2 in cytoplasm was reduced by 50% (Figures 5D–F), which supported the hypothesis that sweroside repressed Keap1 and promoted nuclear translocation of Nrf2.

### The Protective Effect of Sweroside on Oxidative Stress and Pyroptosis Was Nrf2-Dependent

**Figure 6:**
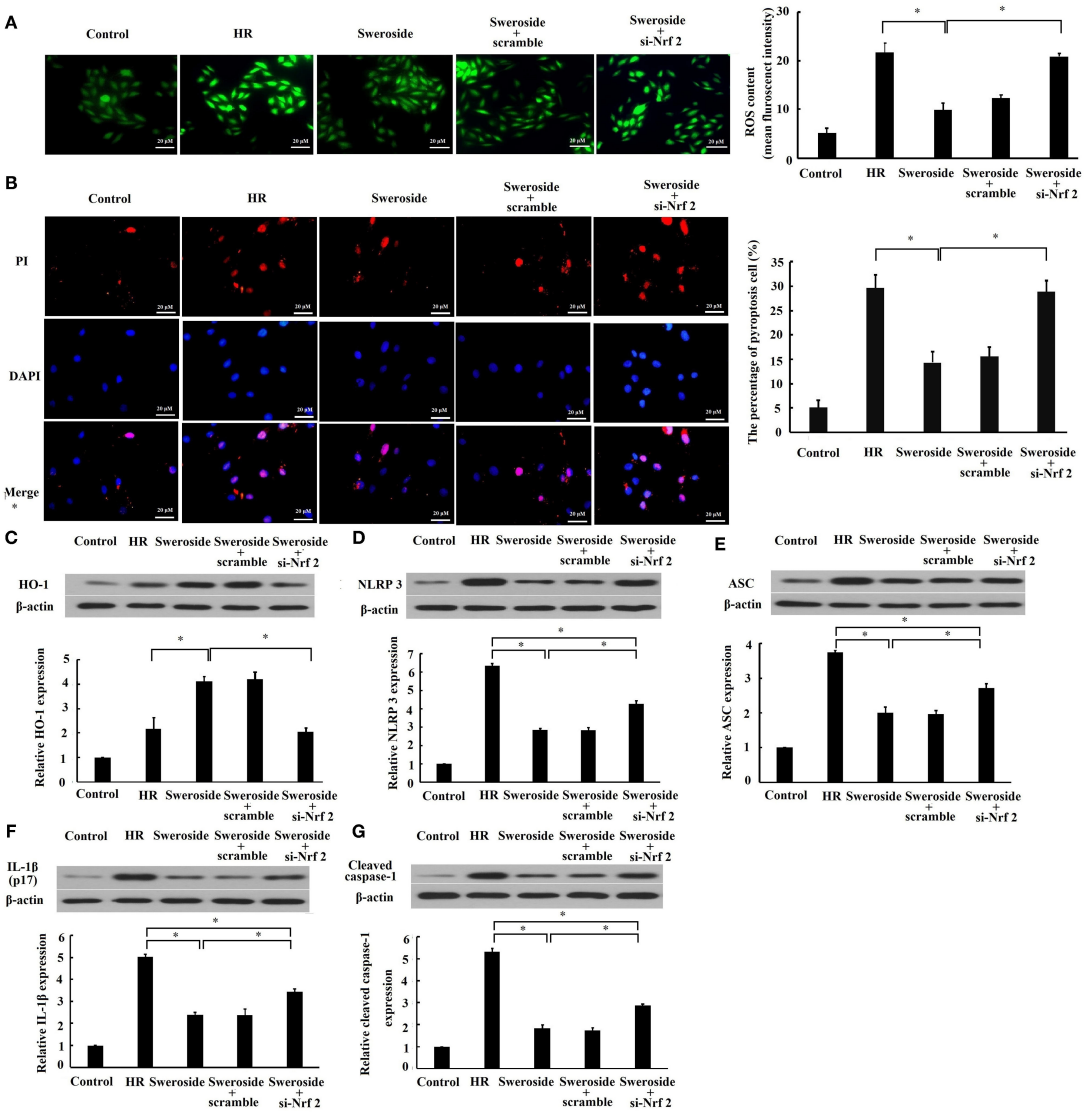
Inhibition of Nrf2 abrogated the effect of sweroside on oxidative stress and pyroptosis. H9c2 cells were transfected with small interfering RNA targeting Nrf2 (si-Nrf2) and its negative control (scrambled siRNA). Following transfection for 24 h, cells were further processed for sweroside treatment and then subjected to hypoxia/reoxygenation (HR). **(A)** Reactive oxygen species (ROS) in cells were detected using 2′,7′-dichlorodihydrofluorescein diacetate (DCFH-DA). **(B)** A pyroptotic cell was characterized by pore formation in the cell membrane. The cell nucleus stained by propidium iodide (PI) indicated a pyroptotic cell. Western blot analysis of the expression levels of HO-1 **(C)**, NLRP3 **(D)**, ASC **(E)**, IL-1β **(F)**, and cleaved caspase-1 **(G)**. Each cell experiment was repeated at least three times. *P < 0.05.

To determine the regulation of Nrf2 in the inhibition of sweroside on oxidative stress and pyroptosis, we examined whether inhibition of Nrf2 by transfection of si-Nrf2 could abrogate the effect of sweroside on oxidative stress and pyroptosis. It was demonstrated that the suppression of ROS and the preservation of cell membrane integrity by sweroside were rescued by inhibition of Nrf2 (Figures 6A,B). Additionally, the enhancement of HO-1 (Figure 6C) was completely rescued by inhibition of Nrf2. However, the repression of NLRP3, ASC, IL-1β, and cleaved caspase-1 expression levels (Figures 6D–G) by sweroside was only rescued 49, 41, 38, and 39% by inhibition of Nrf2, respectively. Taken together, these results suggested that the effect of sweroside on oxidative stress was Nrf2-dependent and its anti-pyroptotic effect was partially Nrf2-dependent.

## Discussion

*Swertia pseudochinensis* Hara, based on its antioxidant and anti-inflammatory activities, has been used to treat hepatitis in China for a long time (27, 28). Sweroside, a main active component of *S. pseudochinensis* Hara, has also been reported to exert multiple pharmacotherapeutic actions. Yang et al. revealed that sweroside alleviated non-alcoholic fatty liver disease in obese mice via the modulation of lipid metabolism and inflammation (29). In addition, Yang et al. indicated that sweroside protected against non-alcoholic steatohepatitis by inhibiting NLRP3 inflammasome-mediated pyroptosis (21). More interestingly, sweroside has been found to prevent myocardial cells against aconitine-induced cardiac toxicity (18). However, the protective effect of sweroside on myocardial IR injury is yet to be determined. To the best of our knowledge, the present study is the first to demonstrate that sweroside could protect against myocardial IR injury, which indicates a potential use for sweroside.

Although a previous study has reported that sweroside could suppress ROS and MDA and enhance SOD activity (18), the specific mechanism for its antioxidant effect is not fully understood. In the present study, we found that sweroside repressed Keap1 expression and subsequently promoted nuclear translocation of Nrf2. Accumulating evidence has revealed that some small molecules could lead to Nrf2 activation by interrupting the Keap1–Nrf2 protein–protein interaction (30, 31). Considering that the interaction between Keap1 and sweroside was predicted in the molecular docking model, we inferred that Nrf2 was activated by interrupting the Keap1–Nrf2 protein–protein interaction by sweroside. Additionally, we confirmed that the effect of sweroside on HO-1 and ROS was rescued by inhibition of Nrf2, which further demonstrated that the antioxidant effect of sweroside was Nrf2-dependent.

Pyroptosis, a new form of programmed cell death, commonly starts with the formation of an inflammasome complex containing NLRP3, and the adaptor proteins ASC and pro-caspase-1 (32). Pro-caspase-1 is cleaved into its active form by the inflammasome complex, and then, on the one hand, active caspase-1 cleaves GSDMD to facilitate the formation of pore at the cell membrane (24), eventually leading to cell lysis (33). On the other hand, the precursors of IL-1β and IL-18 are cleaved by active caspase-1 to produce active IL-1β and IL-18. The inflammatory response is induced when active IL-1β and IL-18 are released out of cells (4). In the present study, we found that sweroside inhibited the expression levels of NLRP3, ASC, IL-1β, and cleaved caspase-1, which suggested that sweroside could repress NLRP3 inflammasome-mediated pyroptosis. This was consistent with findings by Yang et al. (21). In the process of IR, ROS largely accumulate in the tissue and induce NLRP3 inflammasome activation (34). Additionally, Nrf2 activation could inhibit NLRP3 inflammasome activity by repressing ROS (35). In the present study, we also found that the inhibition of NLRP3 inflammasome-mediated pyroptosis by sweroside could be partially reversed by inhibition of Nrf2. This finding suggested that the inhibition of pyroptosis by sweroside may be partially regulated by Nrf2/ROS/NLRP3 axis. Admittedly, this reversion was partial rather than complete, which suggested that other potential pathways might be involved in the anti-pyroptotic effect of sweroside. For instance, NF-κB signaling pathway also plays a key role in mediating the activation of NLRP3 inflammasome-mediated pyroptosis (36, 37). Sweroside is reported to suppress NF-κB signaling pathway (19, 20). Thus, sweroside may inhibit the activation of NLRP3 inflammasome-mediated pyroptosis mediated by NF-κB signaling pathway. However, due to lack of direct experimental evidence for this speculation, additional experiments need to be done to define the strongest/the most potential pathways of sweroside on its anti-pyroptotic effect in the future study.

The limitation of *ex vivo* model established by the Langendorff system is that it cannot completely mimic the pathos-physiological changes during IR due to lack of neurohumoral regulation. However, the isolated heart model has an advantage in control of lots of the variables influencing IR as well as real-time analysis of the functional changes on the heart (38). Although the elimination of systemic influence should be accounted for analyzing our data, we still gain some invaluable results in understanding the cardioprotection induced by sweroside pretreatment.

In conclusion, our study demonstrated that sweroside pretreatment could protect against myocardial IR injury by inhibiting of oxidative stress and NLRP3 inflammasome-mediated pyroptosis at least partially via modulation of the Keap1/Nrf2 axis.

## Data Availability Statement

The raw data supporting the conclusions of this article will be made available by the authors, without undue reservation.

## Ethics Statement

The animal study was reviewed and approved by the Institutional Animal Care and Use Committee of China Medical University.

## Author Contributions

JL, NW, and CM: conceptualization, methodology, software, and validation. JL, CZ, QZ, YW, GL, XL, YL, and NW: formal analysis, investigation, and visualization. CZ, QZ, YW, GL, XL, and YL: resources. JL, CZ, QZ, YW, GL, XL, and YL: data curation. JL and NW: writing—original draft preparation. NW and CM: writing—review and editing, supervision, project administration, and funding acquisition. All authors contributed to the article and approved the submitted version.

## Conflict of Interest

The authors declare that the research was conducted in the absence of any commercial or financial relationships that could be construed as a potential conflict of interest.
